# Insight into eco-epidemiological traits of European native vector mosquitoes for disease transmission

**DOI:** 10.1186/s13071-026-07531-w

**Published:** 2026-07-24

**Authors:** Oliver Chinonso Mbaoma, Stephanie Margarete Thomas, Andrew J. Macdonald, Carl Beierkuhnlein

**Affiliations:** 1 Department of Biogeography, University of Bayreuth, Bayreuth, Germany; 2 Bayreuth Center for Ecology and Environmental Research BayCEER, University of Bayreuth, Bayreuth, Germany; 3 Bren School of Environmental Science and Management, University of California, Santa Barbra, USA

**Keywords:** Native vector mosquitoes, Climate change, *Aedes*, *Culex*, *Anopheles*, Mosquito-borne disease

## Abstract

Transmission risk of emerging and re-emerging mosquito-borne pathogens of major public health concern is increasing in countries at higher latitudes. Although considerable attention has been devoted to understanding how globalization and environmental change influence the epidemiology of diseases transmitted by invasive mosquito species, infections vectored by native mosquito populations have continued to increase drastically. In this Primer, we synthesize current evidence on native mosquito species identified as competent vectors of pathogens in Europe. We explore their key eco-epidemiological traits that support the introduction, establishment, and sustained transmission of pathogens under changing climatic and environmental conditions. Specifically, we provide an overview of their geographic distribution, habitat preferences, host-seeking behavior, host preferences, overwintering characteristics, and competence for disease transmission. Research advances related to changes in habitat use, the consequences of hybridization for pathogen transmission across habitat gradients, and the detection of non-endemic pathogens in field-caught secondary vector species were explored and presented. Additionally, we identify the need for improved delineation of the ecological ranges for important native mosquito vectors, better characterization of the roles of key mosquito species in the current epidemiological landscape, and an enhanced understanding of critical life traits for several important mosquito vectors native to Europe as areas with significant research gaps. Addressing these gaps is essential to ensure enhanced surveillance and improved interventions aimed at mitigating the expanding threat of mosquito-borne diseases in Europe.

**Graphical Abstract:**

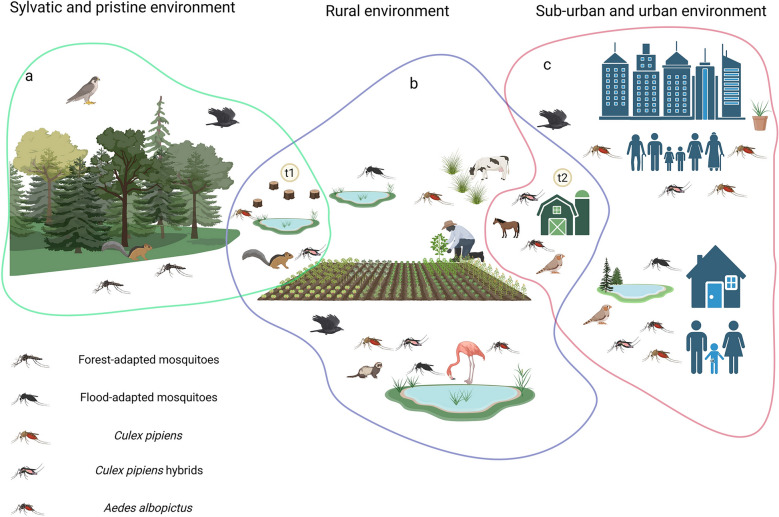

## Background

**Fig. 1:**
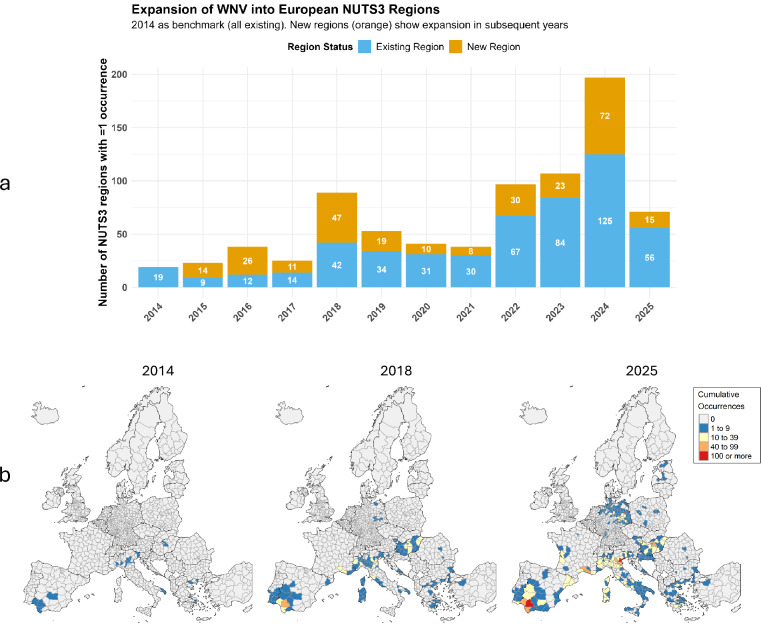
Intensification and spatial expansion of West Nile virus (WNV) infection in horses and birds across Europe. **a** Spatial distribution of WNV cases, expressed as the number of reported cases per NUTS3 region for the years 2014, 2018, and 2025, with number of existing regions infected shown in blue section of the bar while number of new regions affected are shown in yellow section of the bars. **b** Expansion of WNV into new NUTS3 regions, showing the cumulative number of regions affected per year. Both panels illustrate both illustrate the sustained transmission intensity and the geographic spread of the endemic arbovirus, highlighting the role of increasing environmental suitability for pathogen transmission by native vector mosquitoes, particularly species of the *Culex* genus, the primary vectors of WNV in Europe. WNV occurrence data were sourced from Plateforme ESA [61]

Transmission risk of emerging and re-emerging mosquito-borne pathogens of significant public health concern is increasing in countries at higher latitudes, with significant attention directed towards invasive *Aedes albopictus* (*Ae. albopictus*) mosquito species and their role in the emergence of non-endemic mosquito-borne diseases due to the epidemiological importance of the pathogens vectored by them [1, 2]. However, despite successful colonization and establishment of stable populations, autochthonous pathogen transmission in Europe by invasive *Ae. albopictus* mosquitoes remains relatively low compared to those vectored by native mosquitoes [3]. According to the European Centre for Disease Prevention and Control (ECDC), since the beginning of 2025, only two European countries (France and Italy) have reported autochthonous cases of chikungunya (CHIKV) infections, while dengue virus (DENV) infections have been recorded in France, Italy, and Portugal [4, 5]. In contrast, West Nile virus (WNV) infections, primarily transmitted by native *Culex* mosquitoes, have been far more widespread, with 1112 locally acquired human cases reported from 14 European countries (Fig. 1) as of 3 December 2025 [6]. The distinction in geographic distribution of WNV from DENV and CHIKV highlights the role of well-adapted status of its primary vector in supporting the emergence, re-emergence, and spatial expansion across regions in Europe (Fig. 1b), with new cases detected in non-endemic locations yearly (Fig. 1).

**Fig. 2:**
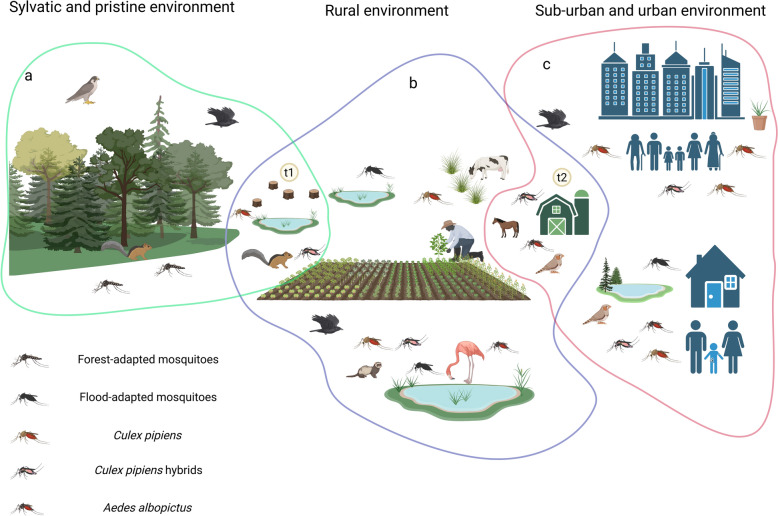
Landscape gradient and its effect on native vector mosquito eco-epidemiology. **a** Sylvatic/pristine environments characterized by enzootic pathogen cycles between mosquitoes and wildlife with minimal human contact. **b** Rural environments associated with fragmented forests, agriculture, and domestic animals increase breeding sites, host interactions, and hybridization, elevating spillover risk. **c** Sub-urban/urban environments where intensified human activity supports transmission by urban-adapted vectors, facilitating outbreaks. Transition zones are indicated as t1 (forest to altered habitat) and t2 (rural to urban). Created in https://BioRender.com

Several studies have provided insight into key eco-epidemiological traits of mosquito vectors well-established and have been involved in disease transmission in Europe or identified as highly competent vectors of pathogens of interest. Eco-epidemiological traits of the selected mosquito vectors presented in this study relates to their occurrence, habitat preferences, host-seeking behavior and host preferences, overwintering strategies, and competence for pathogen transmission [7–9]. Given the changing epidemiological landscape of mosquito-borne diseases in Europe (Fig. 2), insight into the characteristics of these traits would be important to support current mosquito-borne disease management and control efforts.

The motivation for exploring existing gaps within the eco-epidemiological traits of mosquitoes in Europe arises from the changing interactive effects of ecological and epidemiological processes under global change. Insight into vectorial competence, which refers to the ability of a mosquito species to successfully acquire, maintain, and transmit a pathogen to a susceptible host [69], is essential for understanding the epidemic landscape of a given location. Spill-over of mosquito-borne pathogens which occurs as a result of a vector feeding on multiple host species across diverse habitat types, enabling it to act as a bridge and facilitate cross-species pathogen transmission, is a key determinant of the epidemiological landscape in a location [68]. Consequently, the role of well-adapted vector species considered native to a region in pathogen transmission is a function of their vectorial competence, habitat preference, and host-seeking behavior, which together determine their capacity to act as primary or bridge vectors and influence transmission dynamics.

In this primer, we present an overview into traits of selected native mosquito species that have been identified in previous field studies as vectors of significant pathogens, experimentally shown to be susceptible to key pathogens, or historically involved in transmitting pathogens that were previously eradicated but are now re-emerging. We also present advances in research on changes relating to certain eco-epidemiological traits and the implications for disease transmission. Finally, we identify three areas ripe for further investigation to improve understanding and support enhanced surveillance, targeted vector control, and more effective disease management.

### Native vector mosquitoes

Several mosquito vectors have been native to Europe for a long time, most of which are well-adapted to local environmental conditions and are widespread across Europe's landscapes.

Mosquito species included in this study were selected based on their ecological adaptability, geographic distribution, and epidemiological relevance in Europe. Species from the genera *Culex*, *Aedes*, and *Anopheles* represent key vector groups due to their widespread occurrence, ability to occupy diverse habitats, and involvement in the transmission of pathogens of public health importance.

We considered *Culex* mosquitoes because they are widely distributed across the northern Hemisphere and are important vectors in Europe due to their ecological plasticity and role in pathogen transmission [3]. Their ability to exploit diverse natural and artificial habitats, combined with hybridization and flexible host-feeding behavior, enhances their role as bridge vectors [32–34, 37].

Native European *Aedes* species were selected based on their broad ecological range, high densities, and potential for pathogen transmission. These species occupy habitats such as agricultural areas, wetlands, and tree holes, and exhibit opportunistic feeding, making them relevant for zoonotic transmission [18–20, 23, 43].

*Anopheles* species were selected based on their role in malaria transmission. Although malaria has been eliminated in Europe, several native species persist, particularly within the *Anopheles maculipennis* complex [62]. Their importance of being designated as a potential vector of malaria in Europe as presented by Bertola et al. [9] classified *Anopheles hyrcanus* s.l., *Anopheles labranchiae*, *Anopheles plumbeus*, and *Anopheles sacharovi* as highly important vectors; *Anopheles messeae/daciae* and *Anopheles maculipennis* s.s. as moderately important; and *Anopheles atroparvus* and *Anopheles superpictus* as of low importance.

**Fig. 3:**
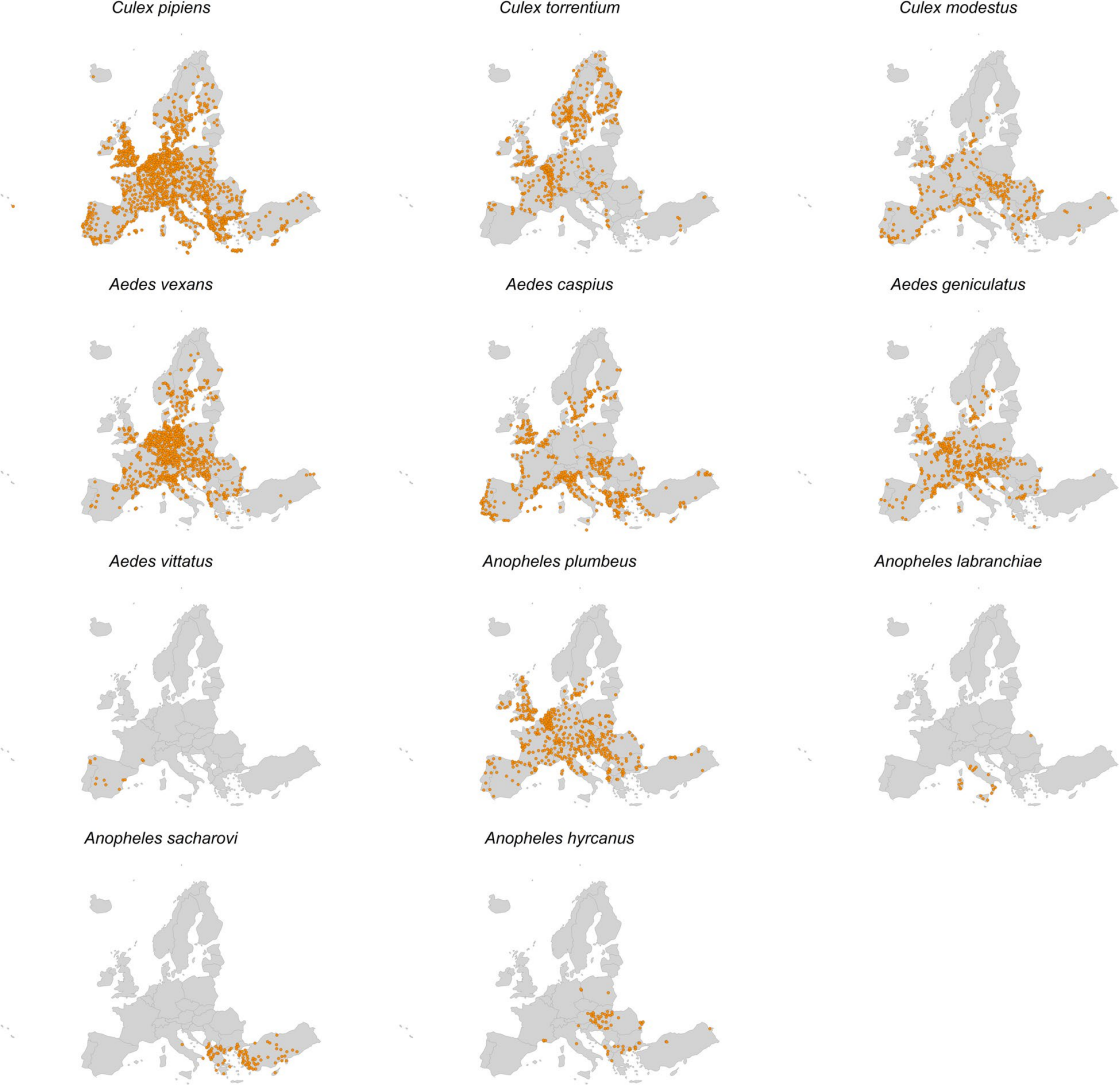
Spatial distribution of vector mosquitoes of interest from the *Culex*, *Aedes* and *Anopheles* genera in Europe extracted from GBIF database

Here, we focused on vector mosquitoes from the *Culex*, *Aedes* and *Anopheles* genera, as they have been shown to contribute to the transmission and spread of various diseases of public health concern. These include species from the genus *Culex*—*Cx. pipiens*, *Cx. torrentium* and *Culex modestus*; from the genus *Aedes*—*Ae. vexans*, *Aedes caspius*, *Ae. geniculatus, Ae. vittatus*; and from the genus *Anopheles*—*Anopheles plumbeus*, *An. hyrcanus*, *Anopheles labranchiae* and *An. sacharovi* (Fig. 3). We also highlighted important characteristics of each species in terms of their distribution, habitat preference, host-seeking behavior and pathogen competence (Table 1).

**Table 1:** Summary of the status of important eco-epidemiological traits of mosquito vectors native to Europe detailing the geographical distribution, habitat preference, host-seeking behavior, and pathogen competence

Species	Distribution	Habitat preference	Host-seeking behavior	Pathogen competence	References
*Cx. pipiens*	Widespread across Europe	Urban/rural, temporal water bodies, containers	Biotype-dependent: ornithophilic or anthropophilic	WNV (field), USUV (field), SLEV, RVFV (lab), *Dirofilaria* spp. (lab and field)	[3, 10, 11]
*Cx. torrentium*	Northern, central Europe and high elevation in the south	Urban, cold shaded wetlands, water containers	Mainly ornithophilic, but also anthropophilic	SINV (field), WNV (lab), USUV	[12–14]
*Cx modestus*	Southern, central Europe, currently expanding northwards	Rural, rice fields, marshes, fishponds, wetlands, deciduous forests	Opportunistic: feeds interchangeably on birds, humans, mammals	WNV (field and lab), *Dirofilaria spp*, *Plasmodium relictum*	[15–17]
*Ae. vexans*	Widespread across Europe, especially floodplains	Temporary flood pools, marshes, and vegetated coastal wetlands	Mammalophilic, aggressive day-biter	WNV (field and lab), TAHV (field), RFVF, ZIKV (lab)	[18, 19]
*Ae. caspius*	Widespread across Europe	Brackish wetlands, rice fields, coastlands marshes, inland marshes, irrigated fields and swamps	Strongly anthropophilic, nuisance species	Dirofilaria spp. (field), WNV (lab), TAHV (field), RVFV (lab), CHIKV (lab)	[20–22]
*Ae. geniculatus*	Widespread across Europe	Tree holes, shaded woodland areas, occasionally in water containers	Mammalophilic; aggressive dusk and dawn feeders	Undocumented field role; potential for CHIKV (lab)	[8, 23]
*Ae. vittatus*	Western -Mediterranean Europe	Natural (rock pools, tree holes) and artificial containers (domestic containers), human-modified habitats	Opportunistic: feeds on humans and livestock	CHIKV, ZIKV (Field), DENV, Yellow fever (lab)	[63–67, 70]
*An. hycanus*	South, eastern and central Europe	Fishponds, swamps, rice fields and floodplains	Mammalophilic	*Plasmodium falciparum*, *Dirofilaria immitis * and *Dirofilaria repens*	[9, 24, 25]
*An. labranchiae*	Mediterranean Europe	Fresh water including pits, canals, drains, flooded pools, lakes	Day-active, most aggressive *Anopheles* species and opportunistic feeders,	*Plasmodium falciparum* (lab)	[9, 26, 27]
*An. sacharovi*	Southeastern Europe, Balkans	Brackish and fresh water, irrigated lands, swamps, rice fields,	Zoophilic and anthropophilic	*Plasmodium falciparum* and *Plasmodium vivax* (historic and lab)	[9, 28, 29]
*An. plumbeus*	Widespread across Europe except far North	Tree holes, containers, ponded rainwater, flower vases and used tires	Opportunistic feeders; day-active nuisance, increasingly anthropophilic	*Plasmodium falciparum* and *Plasmodium vivax* (lab)	[2, 9, 30, 31]

### *Culex* species

*Cx. pipiens* Linnaeus, 1758 (*Cx. pipiens*) is the most widely distributed mosquito vector in Europe [11]. They consist of two biotypes, which are *Cx. pipiens* f. pipiens and *Cx. pipiens* f. molestus and their hybrids, which are morphologically similar but ecologically distinct, breeding across diverse natural and urban habitats [10]. *Cx. pipiens* f. molestus is predominantly anthropophilic and urban, reproducing year-round in subterranean environments, while *Cx. pipiens* f. pipiens are primarily ornithophilic, common in peri-urban areas, adapted to colder climates, and undergoes diapause [32, 33]. *Cx. pipiens* SSL hybrid forms are commonly detected at urban–rural interfaces, exhibit opportunistic feeding by occasionally taking blood meals from birds, mammals and humans making them efficient bridge vectors [32–34]. Globally, *Cx. pipiens* have been involved in the transmission of numerous pathogens, including WNV, Usutu (USUV), St. Louis encephalitis virus (SLEV), Western and Eastern equine viruses (WEEV, EEEV), filarial worms and avian malaria [35]. In Europe, *Cx. pipiens* populations and hybrids are key vectors of WNV and USUV, with experimental competence demonstrated for Rift Valley fever virus (RVFV) and Japanese encephalitis virus (JEV) [3, 36].

*Cx. torrentium* Martini, 1925 (*Cx. torrentium*) occurs across much of Europe, with a preference for colder regions of northern and central Europe although its precise range remains uncertain [12, 37]. Morphologically quite similar to *Cx. pipiens*, the two species often co-occur, particularly in urban container habitats [37, 38]. *Cx. torrentium* feeds on birds, mammals, and humans, making it a potential bridge vector [13]. They are considered the primary vectors of Sindbis virus (SINV) in Europe and have been shown experimentally to be more competent for WNV than *Cx. pipiens* f. pipiens [12, 39]. Field and laboratory evidence also support their role as vectors of USUV [40, 41].

*Culex modestus* Ficalbi, 1890 (*Cx. modestus*) is distributed throughout the Palearctic region, with European populations concentrated in southern and central areas [15, 42]. Unlike the ecologically plastic *Cx. pipiens*, *Cx. modestus* populations are largely restricted to rural and agricultural habitats associated with permanent or semi-permanent water bodies, including rice fields, marshes, and fishponds [16]. Although often described as ornithophilic, it is an opportunistic feeder that readily bites humans and other mammals, supporting their role as bridge vectors [16, 17]. They have been identified as important vectors of WNV in Spain and has demonstrated vectorial potential for USUV, *Dirofilaria immitis*, and *Plasmodium relictum* [16, 17].

### *Aedes* species

*Aedes vexans* (*Ae. vexans*) mosquito species are widely spread across the Holarctic region [18]. They prefer to oviposit in floodplains close to river and vegetated coastal wetlands with the presence of trees, bushes and reeds [19]. Their populations can increase rapidly following flooding events, with their dormant eggs capable of surviving adverse conditions for several years [43]. The species is strongly anthropophilic and mammaliophilic, feeding readily on humans and large mammals, which supports its role in bridging pathogen transmission between animal reservoirs and humans [18]. In Europe, *Ae. vexans* are considered both a major nuisance and a competent vector of several arboviruses, including WNV, RVFV, and Tahyna virus (TAHV) [18, 44]. WNV was recently isolated from field caught *Ae. vexans* in the United Kingdom [44].

*Aedes caspius* Pallas, 1771 (*Ae. caspius*) is a floodwater mosquito widely distributed across Europe and an important nuisance species [20, 45]. They have diverse habitat preferences which include rice fields, coastlands marshes, inland marshes, irrigated fields, and swamps [45]. Modern agricultural practices have been implicated to increase the availability of breeding sites and support their population expansion recently [7]. They actively seek hosts during dusk and dawn and are reportedly aggressive feeders while taking bloodmeals from humans and other mammals [22, 45]. *Ae. caspius* is a competent vector of TAHV and RVFV, a potential vector of chikungunya virus, and has low competence for Zika virus (ZIKV) [10, 46]. They have also been implicated in the transmission of *Dirofilaria immitis* and *Dirofilaria repens* [21].

*Aedes geniculatus* Olivier, 1791 (*Ae. geniculatus*) mosquitoes are primarily associated with tree-hole habitats in deciduous and mixed forests. However, they can also exploit artificial containers and are commonly detected in ovitraps used to sample invasive *Ae. albopictus* [8]. Their eggs can overwinter and survive desiccation [47]. The species is an aggressive dusk and dawn-active biter, feeding mainly on mammals, including humans and livestock [47]. Although not considered a major vector in nature, *Ae. geniculatus* has been shown experimentally to be highly susceptible to chikungunya virus infection and transmission [8].

*Aedes vittatus* Bigot, 1861 (*Ae. vittatus*) is a mosquito species found in southern Europe and typically associated with habitats in urban, rural and peri-urban environments [63, 66, 70]. They breed in diverse natural and artificial habitats, including rock pools, tree holes, and domestic containers, with larvae detected in both natural and human-modified sites, reflecting high ecological plasticity and strong adaptation to anthropogenic environments [66, 67]. *Ae. vittatus* is an opportunistic feeder on humans and livestock (bovids, sheep/goats, porcupines), supporting its role as a potential bridge vector [63, 67]. *Ae. vittatus* is a confirmed vector of several arboviruses including chikungunya and Zika [65, 67], and a potential vector of dengue and Yellow fever [64] highlighting its epidemiological relevance.

### *Anopheles* species

*An. hyrcanus* Pallas, 1771 (*An. hyrcanus*) is distributed across the Palearctic and Oriental regions and occurs in southern, eastern, and central Europe [9]. They breed primarily in large stagnant water bodies, including rice fields, swamps, fishponds, and floodplains [9, 48]. They are mainly mammaliophilic, feeding on large mammals and humans, and are considered important vectors in several European regions [24, 25]. They were designated as the most important potential malaria vector in France and were also isolated to have played a role in malaria transmission in Afghanistan [48, 49]. They are also potential vectors for important parasitic pathogens like *Dirofilaria immitis* or *Dirofilaria repens* [25].

*Anopheles labranchiae* Falleroni, 1926 (*An. labranchiae*) are primarily found in Mediterranean Europe [9]. They breed mainly in rice fields and other freshwater habitats with preference for slow-moving water, canals, drains, and flooded pools [26, 27]. Recent increase in their population has been linked to the reintroduction and intensification of rice cultivation [27]. They are considered the most aggressive *Anopheles* mosquito species during host-seeking and are mainly opportunistic feeders readily feeding on humans and animal hosts [9, 26]. They can occasionally seek blood meals without ovipositing making them non-gonoactive feeders and shaping their vectoral potentials [26]. *An. labranchiae* has been reported to be susceptible and could also potentially transmit tropical strains of *Plasmodium falciparum* experimentally [27].

*Anopheles plumbeus* Stephens, 1828 (*An. plumbeus*) mosquitoes are widely distributed across Europe, excluding the far north [2, 30]. Historically associated with tree-hole breeding, they are increasingly colonizing artificial containers such as septic tanks, catch basins, and tires [31]. They are aggressive, day-active biting mosquitos and significant nuisance species. Besides humans, they have exhibited a preference to feed on horses, cattle and birds occasionally, making them potential bridge vectors of pathogen [31]. Laboratory studies have confirmed its competence for *Plasmodium falciparum* and *Plasmodium vivax*, and it has been implicated in autochthonous malaria cases in Germany [2, 9].

*An. sacharovi* Favre, 1903 (*An. sacharovi*) occurs mainly in southern Europe and has recently re-emerged in parts of Italy after decades of apparent absence [9, 50]. The species exhibits ecological plasticity, tolerating high temperatures and breeding in both fresh and brackish water, though it is sensitive to organic pollution [9, 51]. Although primarily zoophilic, *An. sacharovi* readily feeds on humans when available, supporting its role in malaria transmission [28]. Historically, they have been major malaria vectors in parts of southern Europe, the Middle East, and western Asia, including documented evidence of *Plasmodium vivax* transmission [9, 29].

### Three advances made in key areas relating to eco-epidemiological traits

#### Changes in habitat preference

Substantial shifts in habitat preference and increasing ecological plasticity have been documented in several mosquito species native to Europe, reshaping their epidemiological relevance. *Cx. pipiens* mosquitoes previously associated with natural and peri-urban habitats, now thrive across a wide range of urban environments (Fig. 2), facilitated by behavioral divergence among its biotypes and their hybrids [52, 53]. Studies have also reported that *Cx. pipiens* populations remained more stable in urban areas compared to peri-urban locations, potentially leading to sustained mosquito-related risk in urban environments [53]. *An. plumbeus* and *Ae. geniculatus*, both historically restricted to tree holes in forested landscapes, has increasingly colonized artificial containers especially in urban areas, including septic tanks and urban catch basins, bringing this competent malaria vector into closer contact with human populations [2, 8, 58].

These habitat shifts enhance human–vector contact and increase the likelihood of pathogen spillover. Similar patterns of habitat flexibility have been reported in mosquito species associated with floodwater and agricultural landscapes. Flood-adapted *Ae. vexans* and *Ae. caspius* have increasingly exploited other anthropogenic waterbodies such as irrigated agricultural landscapes and managed wetlands (Fig. 2), an attribute which functionally extends their habitat beyond natural floodplain, with modern land-use practices amplifying their population densities [21]. Similarly, *An. hyrcanus* and *An. labranchiae* have benefited from the expansion and reintroduction of rice cultivation enabling their population growth and geographic spread, including reappearance in areas where they were previously absent [9, 27, 54]. These reports illustrate how evolutionary shifts in habitat preference, driven by climate change, agricultural intensification, and urbanization, have facilitated the persistence and resurgence of native European mosquito species, with direct implications for the transmission dynamics of arboviral and parasitic diseases.

#### The influence of hybridization in pathogen transmission

Findings related to the impact of hybridization mostly seen in mosquitoes occupying edge habitats (Fig. 2) was an important milestone in research related to understanding epidemiological dynamics of mosquito-borne diseases, spillover effect and epidemic outbreaks especially in zoonotic mosquito-borne diseases [13, 55]. Studies have reported that peri-urban zones characterized by agricultural land use and fragmented landscapes with diverse breeding habitats facilitate the co-occurrence of mosquito populations with divergent ecological traits, thereby increasing opportunities for interbreeding and genetic exchange, as observed in *Cx. pipiens* [33, 56]. Hybridization in *Cx. pipiens* species complex occurs when biotypes interbreed and produce hybrids that exhibit distinct host-seeking behavior different from paternal biotypes exhibiting opportunistic host-seeking making them feed on avian and mammalian hosts interchangeably enhancing their capacity to act as bridge vectors for several zoonotic pathogens such as WNV and Usutu [13, 55, 57]. Additionally, the ability of *Cx. pipiens* hybrids to exploit a broader range of habitats than their parental forms reduce ecological segregation and enhances their role as bridge vectors, thereby increasing pathogen transmission potential by facilitating contact between enzootic and spillover host communities [34].

#### Pathogen detection from secondary vectors in non-endemic regions

Recently, endemic pathogens have been detected in secondary vectors among native mosquito species in regions where these pathogens were previously absent, reflecting ongoing shifts in disease epidemiology. Notably, WNV was recently identified in field-caught *Ae. vexans* specimens in the United Kingdom, marking a significant shift from previous assumptions, as vector competence had previously been demonstrated only under controlled laboratory conditions [44]. This finding is particularly important given that *Ae. vexans* is widely distributed across Europe, occurs in high population densities, and produces desiccation-resistant eggs capable of remaining dormant for several years [43].

### Three areas ripe for further research

#### Ecological range of several species remains uncertain

Despite advances in understanding the ecology of native European mosquitoes, the distribution of several species remains poorly characterized. *Cx. torrentium*, a competent vector of WNV and the primary vector of SINV in Europe, still has an uncertain geographic range due to cryptic morphology and often wrongly classified as *Cx. pipiens* [37]. Although understood to be limited to the Mediterranean [70], the ecological range of *Ae vittatus* in Europe may be underreported due to limited surveillance. Similarly, *Ae. geniculatus* and *An. plumbeus*, which have shifted from tree-hole breeding to artificial urban containers, may be under-documented in rapidly urbanizing and fragmented landscapes. *An. sacharovi*, which has re-emerged in parts of southern Europe after decades of apparent absence, and *An. hyrcanus*, whose populations are linked to changing agricultural practices, may also be more widespread than current records suggest. In addition, species associated with poorly surveyed habitats such as forests and floodplains may be likely underrepresented in existing surveillance data, highlighting possible gaps in entomological surveillance across Europe.

#### Uncertainty about pathogen transmission in several native species

The role of certain mosquito species in endemic pathogen transmission remains poorly defined. For instance, *Cx. torrentium* has long been speculated to contribute to WNV and USUV transmission in Europe, yet its role remains unclear due to difficulties in morphologically distinguishing it from *Cx. pipiens* [41]. This situation contrasts with what is obtainable in southern Europe, where *Cx. modestus* and *Cx. pipiens* have been clearly identified as the primary vectors of WNV [31, 58], enabling more targeted and efficient vector control efforts. Although several studies have documented *Ae. vittatus* as competent vector for a range of pathogens in non-European regions [64, 65, 67], there is currently no empirical evidence assessing the vector competence of European populations of this species representing a critical knowledge gap. Also, while the epidemiological importance of *Cx. modestus* has been well documented in southern Europe, its role in central and eastern Europe remains less explored despite studies reporting isolating important pathogens such as WNV and SINV RNA in field collected specimen in Romania [59], and in regions where it co-occurs with multiple competent *Culex* species [15]. Similarly, *Ae. vexans* have an unclear role in sustained arbovirus transmission despite being widely distributed across Europe. Although laboratory studies have demonstrated its competence for several viruses, including WNV and RVFV, field-based evidence is limited, leaving its contribution to enzootic or epidemic transmission cycles poorly resolved. The recent detection of WNV in field-caught *Ae. vexans* from a previously non-endemic location (United Kingdom) underscores a changing epidemiological landscape [44] and signals the need to intensify surveillance and pathogen screening of competent native mosquito species to better understand their evolving roles in disease transmission.

#### Life traits of several species remain uncharacterized experimentally

Lastly, limited knowledge of the life traits of several native mosquito species of epidemiological importance constrains the application of dynamic ecological modeling approaches that could complement traditional surveillance methods. While key traits of *Cx. pipiens* and *Ae. vexans* have been well characterized through experimental studies, life trait parameters for other important species, including *Cx. torrentium*, *Cx. modestus*, and several others, remain poorly documented. This lack of species-specific ecological data hinders the development of robust, ecologically informed models suitable for early warning systems and limits the ability to accurately predict transmission risk under changing environmental conditions, especially in under-sampled locations. To shed light into the risk associated with under-sampled locations, a recent study conducted in an under-sampled location of the central Balkans, WNV, *Dirofilaria immitis*, *Dirofilaria repens*, *Setaria tundra*, *Trypanosoma* spp. and *Plasmodium* spp. were detected in field-caught *Cx. pipiens/torrentium*, underscoring gaps in surveillance efforts for characterizing vector diversity and pathogen transmission potential which may exist in several hotspots in Europe [60].

## Conclusions

Substantial progress has been made in understanding eco-epidemiological traits of native European mosquito vectors including shifts in habitat use, hybridization effects across habitat gradients, and the emergence of non-endemic pathogens in field-caught secondary vector species, but major knowledge gaps persist. More precise delineation of the ecological ranges would improve the efficiency of surveillance and control strategies. Clearly identifying species-specific roles of key mosquito species is essential for accurate monitoring and management of mosquito-borne diseases. Finally, better experimental characterization of key-life-traits in several important vector mosquitoes native to Europe would support the development of robust ecological models and strengthen the capacity of existing surveillance methods, particularly in under-sampled regions.

## Data Availability

References to all data used for this research have been provided in-text.
